# Serum procalcitonin has no significance in the diagnosis of periprosthesis joint infection before total hip and knee replacement

**DOI:** 10.3389/fsurg.2023.1216103

**Published:** 2023-11-06

**Authors:** Xiaobo Sun, Haitao Zhang, Yuting Liu, ZhiWei Lai, Yirong Zeng

**Affiliations:** ^1^ Fourth Orthopedic Department, Ganzhou Hospital of Traditional Chinese Medicine, Ganzhou, China; ^2^ The First Clinical Medical School, Guangzhou University of Chinese Medicine, Guangzhou, China; ^3^ Academic Affairs Office, Gannan Medical University, Ganzhou, China; ^4^ Department of Orthopaedics, The First Affiliated Hospital of Guangzhou University of Chinese Medicine, Guangzhou, China

**Keywords:** periprosthetic joint infection, total joint arthroplasty, serum procalcitonin, complication, diagnostic performance

## Abstract

**Background:**

Currently, there is no “gold standard” for early diagnosing PJI. The diagnosis of periprosthetic joint infection (PJI) is a challenging problem in the clinic. As we know, many serum markers have been used in the early diagnosis of PJI. The aim of this study was to validate the value of PCT in the diagnosis of PJI.

**Methods:**

A retrospective review of 77 patients with revision arthroplasties from January 2013 to July 2020 was conducted. PJI was defined using the modified Musculoskeletal Infection Society (MSIS) criteria combined with follow-up results. Besides medical history, clinical and laboratory data was gathered. Preoperative blood was taken for serum PCT and other biomarkers measurement. Receiver operating characteristic (ROC) curves were generated to evaluate the biomarkers’ diagnostic performance and optimal cut-off value.

**Results:**

Forty-one patients were identified as the PJI group (27 hips and 14 knees), while thirty-six patients were identified as the aseptic loosening (AL) group (33 hips and 3 knees). The AUCs for C-reactive protein (CRP), erythrocyte sedimentation rate (ESR), Platelets (PLT), Fibrinogen (FIB), and Procalcitonin (PCT) were 0.845 (95% CI 0.755–0.936, *p* < 0.001), 0.817 (95% CI 0.718–0.916, *p* < 0.001), 0.728 (95% CI 0.613–0.843, *p* < 0.001), 0.810 (95% CI 0.710–0.910, *p* < 0.001) and 0.504 (95% CI 0.373–0.635, *p* = 0.950), respectively. Higher Area under the Curve (AUC) values were obtained for the combinations of PCT and CRP (AUC = 0.870) (95% CI, 0.774–0.936), PCT and ESR (AUC = 0.817) (95% CI, 0.712–0.896), PCT and PLT (AUC = 0.731) (95% CI, 0.617–0.825), PCT and FIB (AUC = 0.815) (95% CI, 0.710–0.894). The serum PCT indicated a sensitivity of 19.51% and a specificity of 83.33% for diagnosing PJI. When the optimal cut-off value for PCT was set as 0.05 ng/ml, its positive and negative likelihood ratios were 57.1% and 47.6%, respectively.

**Conclusion:**

In conclusion, serum PCT appeared to be no reliable biomarker in differentiating PJI from aseptic loosening before revision arthroplasties. However, PCT combined with other biomarkers further increases the diagnostic accuracy.

## Introduction

Total joint replacement (TJA) is the most effective treatment for advanced arthritis, but periprosthetic joint infection (PJI) is a serious complication and a major reason for postoperative revision (1, 2). The incidence of PJI after arthroplasty was 0.7% (3). A study has shown that each patient suffered from PJI would pay at least $15,000–$30,000 (4). Therefore, early diagnosis of PJI and effective intervention are very to improve the prognosis after total joint replacement. A large number of clinical studies have shown that early diagnosis can not only preserve the prosthesis to a certain extent, but also the infection control rate can reach 70% (5). Currently, there is no “gold standard” for early diagnosing PJI. The diagnosis of periprosthetic joint infection (PJI) is a challenging problem in the clinic. This leads to the delayed diagnosis and treatment of PJI. As a result, this catastrophic complication can seriously diminish patient quality of life and increase the financial burden on families and societies (2).

Serum samples are readily available and are especially important for patients without Synovial fluid samples. As we know, many serum markers have been used to diagnose PJI early. Erythrocyte sedimentation rate (ESR) and C-reactive protein (CRP) are recommended as essential indicators for the diagnosis of PJI by the American Academy of Orthopaedic Surgeons (AAOS) and the Musculoskeletal Infection Society (MSIS) guidelines due to their superior sensitivity and specificity (6, 7). Zhang et al. ﬁrst reported that serum platelet (PLT) is a promising marker for the diagnosis of deep surgical site infection after open induction internal fixation for traumatic limb fractures (8). Klim, S.M. et al. showed that Fibrinogen (FIB) is a cost-efficient and practical marker for diagnosing PJI (9, 10). Procalcitonin (PCT) is commonly used for the diagnosis of systemic infection (11–13). However, its diagnostic value for PJI is controversial, and there is no universal threshold value (14–16). To further clarify its diagnostic value, this study evaluated the value of PCT in the diagnosis of PJI by comparing it with CRP, ESR, PLT, and FIB.

In this retrospective study, we sought to: (1) the performance of PCT in distinguishing chronic PJI and AL (Aseptic Loosening) by comparing with other inflammation indicators; (2) the value of PCT combined with CRP, ESR, PLT, or FIB for diagnosing PJI.

## Methods

### Study design

After approval of our hospital's institutional review board, a single-center retrospective cohort study protocol was performed in compliance with the Helsinki Declaration. We recruited patients after revision hip or knee arthroplasties from January 2013 to July 2020 in our institution to determine the diagnostic value of PCT for diagnosing PJI.

### Inclusion and exclusion criteria

**Figure 1:**
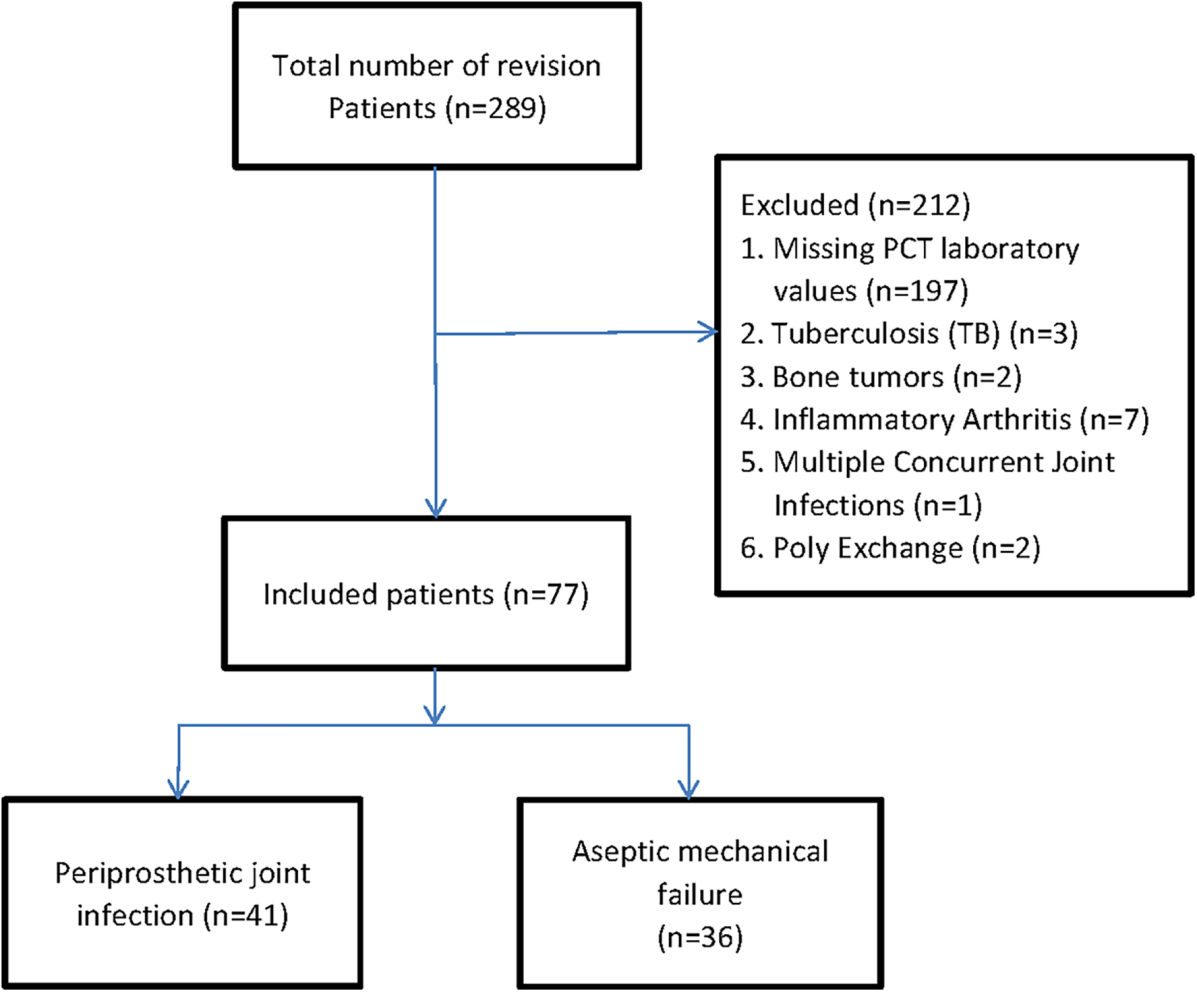
Flow diagram of patients shows the study design.

We identified patients with revision hip or knee arthroplasties using the International Classification of Diseases, Tenth Revision, and Clinical Modification procedure codes (17). A total of 289 patients with revision arthroplasties were originally included into our retrospective cohort study. The primary causes of joint revision and clinical symptoms before surgery are pain, systemic or local joint fever, joint swelling or sinus formation, etc. Firstly, patients without serum PCT at revision arthroplasties were excluded. In order to diminish the possibility of bias associated with comorbidities, we excluded 12 patients with the history of tuberculosis (TB) (*n* = 3), bone tumors (*n* = 2), and inflammatory arthritis (*n* = 7). And those patients who had undergone multiple concurrent joint Infections (*n* = 1), Poly exchange surgery (*n* = 2) also were excluded due to its intricate source of pathogens and undetermined duration of infection (18). Finally, 77 patients were included in the analysis, which was divided into two groups: 41 patients in the periprosthetic joint infection group (PJI group) and 36 patients in the aseptic loosening group (AL group) (Figure 1). Patient's age, gender, and other baseline data were compared between the two groups (Table 1).

**Table 1:** Demographic data for the study population.

Characteristic and group	PJI	AL	*p*-value	*Z*-value
	(*n* = 41)	(*n* = 36)		
Mean age, years (SD)	64.5 (10.0)	65.9 (9.8)	0.529^†^	
Sex, *n* (%)			0.321^‡^	
Male	17 (41.46%)	11 (31.56%)		
Female	24 (58.54%)	25 (69.44%)		
Joint type, *n* (%)			0.014^§^	
Hip	27 (65.85%)	33 (91.67%)		
Knee	14 (34.15%)	3 (8.33%)		
Mean Time1, years (quartile)	3.0 (1.0–5.5)	8.0 (3.3–13.5)	0.000*	−3.61
Mean Time2, months (quartile)	5.0 (1.0–9.5)	10.0 (3.3–24.0)	0.001*	−3.31
Diabetes mellitus, *n* (%)			0.094^§^	
Diabetic	7 (17.07%)	1 (2.78%)		
Nondiabetic	34 (82.93%)	35 (97.22%)		
Blood pressure, *n* (%)			0.683^‡^	
Hypertension	13 (31.71%)	13 (36.11%)		
Nonhypertension	28 (68.29%)	23 (63.89%)		

PJI, periprosthetic joint infection; AL, aseptic loosening; *n*, sample size; SD, standard deviation; Time1, time from primary arthroplasty to the first reoperation (years); Time2, time from symptom onset to the first reoperation (months).

*P* < 0.05 was regarded as statistically significant.

* Independent-samples nonparametric test (Mann–Whitney *U*).

^†^ Independent-samples *t*-test.

^‡^ Pearson chi-square test.

^§^ Continuity correction chi-square test.

### Diagnostic criteria of infection and data extraction

The final diagnosis of PJI was based on MSIS criteria (Table 2) (6, 7). Using patients' electronic medical records, we carefully extracted the following baseline data: demographic information, diagnoses, treatments, the involved joint, symptoms and signs, time from primary arthroplasty to the first reoperation (years), time from symptom onset to the first reoperation (months), laboratory results, culture results, comorbidities, and medication use.

**Table 2:** MSIS criteria for the diagnosis of PJI.

	Diagnostic criteria
Major criteria	1) Two positive periprosthetic cultures with phenotypically identical organisms
	2) A sinus tract communicating with the joint
Minor criteria	1) Elevated serum C-reactive protein (CRP > 10 mg/L) AND erythrocyte sedimentation rate (ESR > 30 mm/h)
	2) Elevated synovial fluid white blood cell (WBC > 3,000 cells/ml) count OR change on leukocyte esterase test strip (+ or ++)
	3) Elevated synovial fluid polymorphonuclear neutrophil percentage (PMN% > 80%)
	4) Positive histological analysis of periprosthetic tissue [>5 neutrophils per high-power field in 5 high-power fields (×400)]
	5) A single positive culture

PJI is present when one of the major criteria exists or three out of five minor criteria exist.

### Laboratory evaluations

Patients' cubital fasting venous blood samples were obtained by nurses the day before revision surgery, routinely. The samples were immediately tested by our hospital's laboratory within 1–2 h for PLT and FIB levels. Nurses also took blood samples for serum PCT, ESR, and CRP evaluation at the same time.

In our hospital, at least 3 tissue culture specimens were collected and cultured for 3–7 days. More than one periprosthetic tissues were selected and sent for biopsy and immediate histological analysis by the chief surgeon during the revision surgeries. After that, Vancomycin or a sensitive antibiotic was used to prevent or treat the infection for 2 weeks after the operation. In addition, rivaroxaban was used to prevent deep vein thrombosis in lower limbs. The follow-up time was at least 1 year.

### Statistical analyses

We analyzed clinical and laboratory values by using basic descriptive statistics. As far as quantitative data is concerned, there were two situations. We used the Independent-samples test to compare continuous variables between the PJI and AL groups for normally distributed continuous data which were shown as mean ± standard deviation (SD). On the other hand, we conducted the Mann–Whitney *U*-test to compare continuous variables between groups for non-normally distributed continuous data which were shown as quartiles. In terms of qualitative data, frequencies, and constituent ratios were evaluated to perform the Pearson chi-square test or Continuity correction chi-square test between the two groups. *P* < 0.05 was considered as statistical significance.

The receiver operating characteristic curves (ROC) were plotted to evaluate the diagnostic performance of each serological marker. The areas under the curve (AUC) and 95% confidence intervals (CI) were calculated via ROC analysis. The AUC values were shown as excellent (0.900–1.000), good (0.800–0.899), fair (0.700–0.799), poor (0.600–0.699), or noneffective (0.500–0.599) (19). Youden's index was used to make sure of the optimal predictive cut-offs for each marker. All data were conducted by SPSS software version 23 and MedCalc Software version 15.0.

## Results

A total of 77 patients were included for the final analysis. According to the MSIS criteria, 41 patients were identified as the periprosthetic joint infection (PJI) group (27 hips and 14 knees). In comparison, 36 patients were identified as the aseptic loosening (AL) group (33 hips and 3 knees). The mean age in the PJI group was 64.5 ± 10.0 years; of them, 17 were men and 24 were women. The mean age in the AL group was 65.9 ± 9.8 years; of them, 11 were men, and 25 were women. The two cohorts did not differ statistically in age (*p* = 0.529) and gender (*p* = 0.321). At the same time, there were no statistically significant differences between groups with diabetes mellitus (*p* = 0.094) or hypertension (*p* = 0.683). However, a statistical significance was shown in joint type (*p* = 0.014), time from primary arthroplasty to the first reoperation (*p* = 0.001), and time from symptom onset to the first reoperation (*p* = 0.001) between the two groups. The characteristics of the recruited patients were depicted in Table 1.

We evaluated the tested markers (CRP, ESR, PLT, FIB, and PCT) for all included patients. All patients in the PJI group had significantly higher values for the four markers (CRP, ESR, PLT, and FIB) compared with the AL group (all *P* < 0.05). Unfortunately, there was no significant difference for PCT between the two groups (*P* = 0.747). The details were shown in Table 3. We also listed the normal ranges for these tested markers in Table 3. As shown in Table 4 for culture organisms in the PJI group, Staphylococcus aureus and Streptococcus agalactiae were the two most common pathogens. We classified these pathogens into two groups (Table 5). There were no significant differences in all tested markers (CRP, ESR, PLT, FIB, and PCT) between the two groups (*P >* 0.05). That is to say, the results of the tested markers had nothing to do with the species of pathogens.

**Table 3:** The tested markers in the two groups.

Variables	Normal laboratory	PJI	AL	*P*-value	*Z*-value
CRP (mg/L) (quartile)	0–8	38.3 (14.2–80.3)	5.1 (2.5–11.2)	0.000*	−5.263
ESR (mm/h) (quartile)	M 0–15; F 0–20	60.5 (33.3–83.8)	20.5 (14.0–36.0)	0.000*	−4.750
Platelet (×10^9^/L) (SD)	100–300	312.7 (79.6)	242.5 (85.3)	0.000^†^	
Fibrinogen (g/L) (quartile)	2.00–4.00	4.9 (3.9–5.5)	3.3 (2.8–3.8)	0.000*	−4.743
PCT (ng/ml) *n* (%)	0–0.05			0.747^‡^	
		Negative: 33 (80.49%)	Negative: 30 (83.33%)		
		Positive: 8 (19.51%)	Positive: 6 (16.67%)		

CRP, C-reactive protein; ESR, erythrocyte sedimentation rate; PCT, procalcitonin; PJI, periprosthetic joint infection; AL, aseptic loosening; n, sample size; SD, standard deviation. M, male; F, female.

*P* < 0.05 was regarded as statistically significant.

* Independent-samples nonparametric test (Mann–Whitney *U*).

^†^ Independent-samples *t*-test.

^‡^ Pearson chi-square.

**Table 4:** Culture organisms in the PJI group.

Culture organisms	No. of patients
Positive	30
Staphylococcus aureus	14
Streptococcus agalactiae	4
Staphylococcus epidermidis	2
Staphylococcus haemolyticus	2
Streptococcus constellation	1
Klebsiella pneumoniae	2
E. coli	2
Pseudomonas aeruginosa	1
Enterococcus faecalis	1
Candida tropicalis	1
Negative	11

PJI, periprosthetic joint infection.

**Table 5:** The inflammatory and PCT markers of the 2 most common pathogens in PJI.

Variables	Staphylococcus aureus	Streptococcus agalactiae	*P*-value
	(*n* = 14)	(*n* = 4)	
CRP (mg/L) (SD)	89.47 (120.38)	118.95 (168.60)	0.696^†^
ESR (mm/h) (SD)	61.43 (37.04)	70.00 (25.88)	0.673^†^
Platelet (×10^9^/L) (SD)	290.79 (52.32)	330.50 (83.08)	0.255^†^
Fibrinogen (g/L) (SD)	4.93 (1.39)	5.45 (2.93)	0.615^†^
PCT (ng/ml) *n* (%)			0.948^‡^
	Negative: 8 (57.14%)	Negative: 3 (75.00%)	
	Positive: 6 (42.86%)	Positive: 1 (25.00%)	

CRP, C-reactive protein; ESR, erythrocyte sedimentation rate; PCT, procalcitonin; PJI, periprosthetic joint infection; *n*, sample size; SD, standard deviation.

*P* < 0.05 was regarded as statistically significant.

^†^ Independent-samples *t*-test.

^‡^ Pearson chi-square.

**Figure 2:**
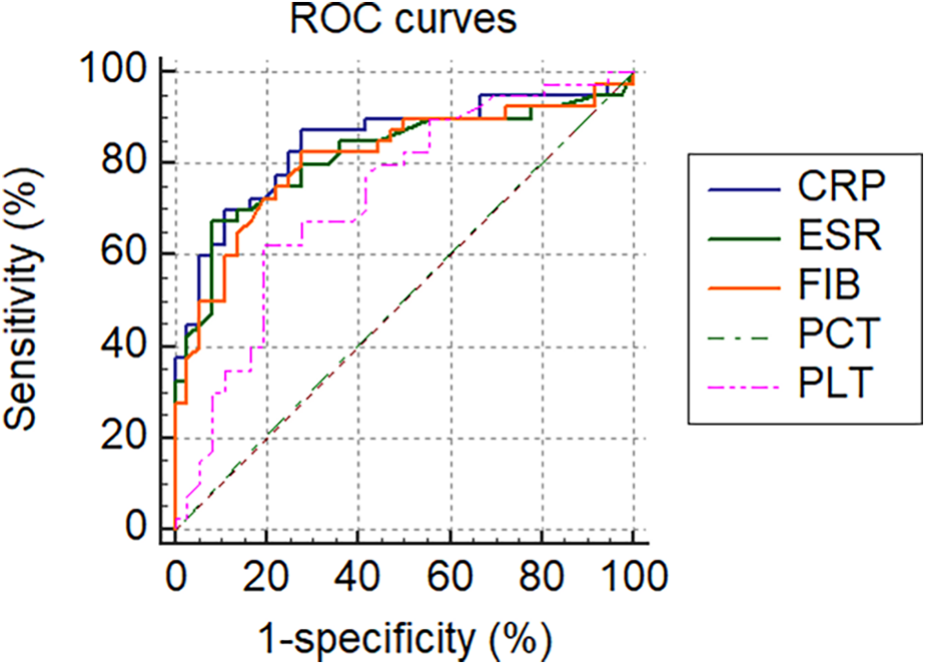
ROC curve of serum markers in diagnosing PJI.

All tested markers (CRP, ESR, PLT, FIB, and PCT) were evaluated and plotted in the ROC curves (Figure 2). The AUCs for CRP, ESR, PLT, FIB, and PCT were 0.845 (95% CI 0.755–0.936, *p *< 0.001), 0.817 (95% CI 0.718–0.916, *p* < 0.001), 0.728 (95% CI 0.613–0.843, *p* < 0.001), 0.810 (95% CI 0.710–0.910, *p* < 0.001) and 0.504 (95% CI 0.373–0.635, *p* = 0.950), respectively (Table 6). The ROC curves showed that CRP had the highest AUC, followed by ESR, FIB, PLT, and PCT. The AUCs of CRP, ESR, and FIB ranged from 0.800–0.899, which demonstrates a good diagnostic value for PJI. The AUC of FIB was between 0.7 and 0.8, indicating a fair diagnostic value for PJI. In contrast, PCT had an AUC of 0.504 (lower than 0.6), the lowest value, indicating an inferior diagnostic value for PJI. Further analyses of the diagnostic value of PCT combined with other markers for PJI were conducted in order to improve their diagnostic accuracies. Higher AUC values indicating better diagnostic accuracy were obtained for the combinations of PCT and CRP (AUC = 0.870) (95% CI, 0.774–0.936), PCT and ESR (AUC = 0.817) (95% CI, 0.712–0.896), PCT and PLT (AUC = 0.731) (95% CI, 0.617–0.825), PCT and FIB (AUC = 0.815) (95% CI, 0.710–0.894). Among them, the combined analysis of PCT with CRP had the highest AUC. In conclusion, combining serum PCT with one of the other markers can improve their diagnostic accuracies (Table 6).

**Table 6:** Area under ROC curve.

Variables	AUC	SE	*P*-value	Asymptotic 95% CI	
				Lower bound	Upper bound
CRP	0.845	0.046	0.000	0.755	0.936
ESR	0.817	0.051	0.000	0.718	0.916
Platelet	0.728	0.059	0.001	0.613	0.843
Fibrinogen	0.810	0.051	0.000	0.710	0.910
PCT	0.504	0.067	0.950	0.373	0.635
PCT and CRP	0.870	0.041	<0.001	0.774	0.936
PCT and ESR	0.817	0.051	<0.001	0.712	0.896
PCT and platelet	0.731	0.058	0.000	0.617	0.825
PCT and fibrinogen	0.815	0.050	<0.001	0.710	0.894

AUC, areas under the curve; SE, standard error; 95% CI, 95% confidence interval (CI); CRP, C-reactive protein; ESR, erythrocyte sedimentation rate.

The serum PCT indicated a sensitivity of 19.51% and a specificity of 83.33% for diagnosing PJI. When the optimal cut-off value for PCT was set as 0.05 ng/ml, its positive likelihood ratio (PPV) and negative likelihood ratio (NPV) were 57.1% and 47.6%, respectively. Based on the ROC analysis, when CRP was above 9.75 mg/L, the sensitivity, specificity, PPV, and NPV were 87.80%, 72.22%, 78.3%, and 83.9%, respectively. Using a cut-off value for ESR of 45 mm/h, the sensitivity, specificity, PPV, and NPV were 67.50%, 91.67%, 90.0%, and 71.7%. Using a cut-off value for PLT at 291 × 10^9^/L, the sensitivity, specificity, PPV, and NPV were 63.41%, 80.56%, 78.8%, and 65.9%, respectively. Using Youden's index, the optimal cut-off value was 3.58 g/L, resulting in sensitivity, speciﬁcity, PPV, and NPV of 82.93%, 72.22%, 77.3%, and 78.1%, respectively (Table 7). From the above results, the value of PCT for diagnosing PJI was inferior to other markers. As shown in Table 7, PCT with the lowest Youden's index (0.02846) has the lowest PLR (1.17) but the highest NLR (0.97). Therefore, the same conclusion can be drawn from Youden's index, positive likelihood ratio (PLR), and negative likelihood ratio (NLR).

**Table 7:** The diagnostic value of tested markers.

Variables	Youden index	Predictive cut-off	Sensitivity (%)	Specificity (%)	PPV (%)	NPV (%)	+LR	−LR
CRP	0.6003	9.75 (mg/L)	87.80	72.22	78.3	83.9	3.16	0.17
ESR	0.5917	45 (mm/h)	67.50	91.67	90.0	71.7	8.10	0.35
Platelet	0.4397	291 (×109/L)	63.41	80.56	78.8	65.9	3.26	0.45
Fibrinogen	0.5515	3.58 (g/L)	82.93	72.22	77.3	78.8	2.99	0.24
PCT	0.02846	0.05 (ng/ml)	19.51	83.33	57.1	47.6	1.17	0.97

CRP, C-reactive protein; ESR, erythrocyte sedimentation rate; PPV, positive predictive value; NPV, negative predictive value; LR, likelihood ratio.

## Discussion

PJI is a catastrophic complication after total joint arthroplasty (20). Currently, there is an international consensus for the diagnosis of PJI, but no “gold standard” (21). The differentiation between PJI and aseptic loosening (AL) is challenging in orthopedic surgery because the treatment of PJI is entirely different to the treatment of aseptic loosening (22). ESR and CRP are initial markers recommended by the current guidelines due to the low false-negative and high sensitivity rates (23–25). CRP is a protein made by the liver. When there is acute inflammation, it responds to increased macrophages (26). Erythrocyte sedimentation rate (ESR) refers to the rate at which red blood cells sink under certain conditions. Although CRP and ESR have shown abilities for diagnosing PJI after primary replacement, the efficacy is limited (27, 28). Research by Paziuk T et al. showed that initial platelet (PLT) counts could be used for distinguishing between PJI and AL (29). Related studies reported that fibrinogen (FIB) with high sensitivity and specificity might become a novel biomarker for diagnosing PJI (19, 30).

PCT, which is produced by Thyroid C cells, consists of 116 amino acids. During infection, serum PCT levels rise with the bacterial endotoxin (31). Therefore, it is helpful to diagnose systemic infection (32). PCT is an undefined biomarker for diagnosing local infections such as PJI (33). The reports about PCT as a marker for the diagnosis of PJI are controversial. Sa-Ngasoongsong et al. showed that PCT was a very specific marker with low sensitivity for diagnosing PJI (34). In contrast, Glehr et al. demonstrated PCT is a sensitive, but not specific marker for diagnosis of PJI (35). Yoon et al. come to a conclusion that PCT is not a promising maker for diagnosing PJI (36). After reviewing reports for the diagnosis of PJI, we found Several studies have illustrated the role of PCT in diagnosing patients with PJI. These studies showed PCT is a sensitive and specific marker of bacterial infection (37–39). We want to investigate whether PCT is a superior maker. Therefore, we performed a sensitivity analysis to further validate the diagnostic performance of PCT. Finally, we found different results which showed the limited efficacy of PCT for diagnosing PJI before revision arthroplasties.

There was no significant difference for PCT between PJI group and the AL group (*P* = 0.747). The AUC for PCT was 0.504 (95% CI 0.373–0.635, *p* = 0.950). The serum PCT indicated a sensitivity of 19.51% and a specificity of 83.33% for diagnosing PJI. When the optimal cut-off value for PCT was set as 0.05 ng/ml, its PPV and NPV were 57.1% and 47.6%, respectively. The results in our study showed that PCT is a specific, but less sensitive biomarker for diagnosing PJI. The AUCs of other biomarkers increased significantly as they were combined with PCT. In conclusion, the combination of serum PCT with one of the other markers can improve their diagnostic accuracies.

Different reasons may result in the limited efficacy of PCT for diagnosing PJI. Firstly, PCT is not necessarily released into the blood if patients suffering from PJI do not show bacteremia (40). It is conceivable that the grade and virulence of the majority of PJI is too low to trigger PCT release. It was pointed out that the high rate of false negatives is associated with local low-virulence organisms (41). Secondly, in healthy adults, even after tooth brushing, transient bacteremia may lead to low-grade PCT release (42–44). Thirdly, since the penetration of PCT into the blood is different in each patient, the cut-off values set for the PCT may affect the study results. The cut-off value (0.05 ng/ml) may not be optimal in our cohort. In addition, we performed only the measurement of serum PCT, while we did not conduct the measurement of synovial fluid PCT.

Some limitations should be considered in our study. Firstly, the modified MSIS criteria used in this study may produce bias for assessing the diagnostic accuracy. Secondly, this was a retrospective cohort study, so its own inherent selection bias may affect its results. Thirdly, only 77 patients were recruited for the study. Thus, a multi-center study with a larger sample size is needed to be carried out for further analyses. This also diminished the sample size. Finally, our study has shown only serum PCT biomarkers, not including synovial fluid PCT biomarkers.

## Conclusions

We detected multiple biomarkers for their diagnostic performance. In conclusion, this study demonstrates that the value of serum PCT has limited efficacy in differentiating PJI from aseptic loosening before revision arthroplasties. However, PCT combined with other biomarkers further increases diagnostic accuracy. Further multiple-center studies with large-size samples are needed to improve its diagnostic rate and validate our results.

## Data Availability

The original contributions presented in the study are included in the article/supplementary material. Further inquiries can be directed to the corresponding author.
